# Unravelling Genetic Factors Underlying Corticobasal Syndrome: A Systematic Review

**DOI:** 10.3390/cells10010171

**Published:** 2021-01-15

**Authors:** Federica Arienti, Giulia Lazzeri, Maria Vizziello, Edoardo Monfrini, Nereo Bresolin, Maria Cristina Saetti, Marina Picillo, Giulia Franco, Alessio Di Fonzo

**Affiliations:** 1 Dino Ferrari Center, Department of Pathophysiology and Transplantation, Neuroscience Section, University of Milan, 20122 Milan, Italy; federica.arienti@unimi.it (F.A.); giulia.lazzeri@outlook.com (G.L.); maria.vizziello@gmail.com (M.V.); edoardo.monfrini@unimi.it (E.M.); cristina.saetti@unimi.it (M.C.S.); 2 Foundation IRCCS Ca’ Granda Ospedale Maggiore Policlinico, Neurology Unit, 20122 Milan, Italy; nereo.bresolin@unimi.it (N.B.); giulia.franco@policlinico.mi.it (G.F.); 3 Center for Neurodegenerative Diseases, Department of Medicine, Surgery and Dentistry, Neuroscience Section, University of Salerno, 84084 Salerno, Italy; mpicillo@unisa.it

**Keywords:** corticobasal syndrome, corticobasal degeneration, CBS, atypical parkinsonism, genetics

## Abstract

Corticobasal syndrome (CBS) is an atypical parkinsonian presentation characterized by heterogeneous clinical features and different underlying neuropathology. Most CBS cases are sporadic; nevertheless, reports of families and isolated individuals with genetically determined CBS have been reported. In this systematic review, we analyze the demographical, clinical, radiological, and anatomopathological features of genetically confirmed cases of CBS. A systematic search was performed using the PubMed, EMBASE, and Cochrane Library databases, included all publications in English from 1 January 1999 through 1 August 2020. We found forty publications with fifty-eight eligible cases. A second search for publications dealing with genetic risk factors for CBS led to the review of eight additional articles. *GRN* was the most common gene involved in CBS, representing 28 out of 58 cases, followed by *MAPT*, *C9ORF72,* and *PRNP*. A set of symptoms was shown to be significantly more common in *GRN*-CBS patients, including visuospatial impairment, behavioral changes, aphasia, and language alterations. In addition, specific demographical, clinical, biochemical, and radiological features may suggest mutations in other genes. We suggest a diagnostic algorithm to help in identifying potential genetic cases of CBS in order to improve the diagnostic accuracy and to better understand the still poorly defined underlying pathogenetic process.

## 1. Introduction

Corticobasal syndrome (CBS) is a rare neurological disorder characterized by a combination of asymmetric akinetic–rigid parkinsonism, fixed dystonic postures, pyramidal signs, and cognitive deficits, such as behavioral changes, speech and language alterations, apraxia, visuospatial impairment, and other cortical signs, including alien-limb phenomena, myoclonus, and cortical sensory loss [1].

Typically, levodopa responsiveness is limited or absent. The clinical features correlate with neuroimaging evidence of asymmetric atrophy and hypometabolism, particularly in the striatum and parietal lobes [2]. CBS has a reported prevalence of 4.9 to 7.3 cases per 100,000 individuals [3]; symptoms usually appear between the fifth and the seventh decade of life [4], and death occurs within 6 or 7 years after the symptoms’ onset [4,5].

Different diagnostic criteria for CBS have been proposed over time, including those advanced by Lang and Bergeron (Toronto) [6], Boeve et al. (Mayo clinic) [7], and Bak and Hodges (Cambridge) [8], as well as the latest by Armstrong et al. in 2013 [9]. Despite extensive efforts in developing more specific diagnostic criteria, the clinical diagnosis of CBS does not always match with neuropathological evidence of corticobasal degeneration (CBD) [10], which is pathologically classified as four-repeat (4R)-tauopathy. In fact, CBD is confirmed in only 54% of clinically diagnosed CBS cases, and the rest show patterns typical of other neurodegenerative disorders, such as progressive Alzheimer’s disease (AD) or supranuclear palsy (PSP) [11]. Conversely, since CBS is a pure clinical entity and not a neuropathological one, it is appropriate to clinically diagnose it in cases with mutations in genes known to be associated with neurodegenerative disorders other than CBD.

CBS is generally recognized as a sporadic disorder, although rare familial and isolated genetic cases have been reported. When genetically determined, CBS has been mainly described in association with mutations in the genes encoding progranulin (*GRN*) [12] or microtubule-associated protein tau (*MAPT*) [12].

The purpose of this paper is to review relevant publications describing cases of genetically determined CBS and to identify distinctive clinical features that may suggest the most likely associated genetic cause.

## 2. Materials and Methods

### 2.1. Literature Search

A systematic review of the literature was performed according to the PRISMA guidelines (Preferred Reporting Items for Systematic Reviews and Meta-Analyses). The search was performed using the PubMed, EMBASE, and Cochrane Library databases and included all publications in English from 1 January 1999 through 1 August 2020.

The following search terms were applied: corticobasal syndrome, corticobasal degeneration, genetic parkinsonism, corticobasal syndrome AND *GRN*, corticobasal syndrome AND *MAPT*, corticobasal syndrome AND *C9ORF72*, and corticobasal syndrome AND *PRNP*.

We performed a second search on genetic risk factors for CBS using as search terms: genetic risk factors for corticobasal syndrome; genome-wide association study AND corticobasal syndrome; *MAPT* H1c haplotype AND corticobasal syndrome; H1/H1 genotype and corticobasal syndrome; *MOBP* AND corticobasal syndrome.

### 2.2. Study Selection and Data Extraction

We included all articles and case reports with a full English text available. Regarding patients’ eligibility, we applied the following inclusion criteria: (1a) clinical diagnosis of possible or probable corticobasal syndrome according to the Armstrong criteria for articles published after 29 January 2013; (1b) clinical diagnosis of corticobasal syndrome according to the Cambridge, Toronto, or Mayo Clinic criteria for articles published before 29 January 2013; (2) positive genetic test results obtained through either Sanger sequencing or Next-Generation Sequencing (exome sequencing or gene panels).

The following exclusion criteria were applied: (1) The clinical description did not fit diagnostic criteria for CBS; (2) the study lacked reliable genetic testing; (3) the original text was unavailable, or the reported information was insufficient; (4) the reported case was presented in a more comprehensive study already considered in the review.

The data extracted from each article included demographic information (age at symptom onset, age at death, and gender), clinical features (both motor and neuropsychological), genetic analysis, and family history of neurological diseases; radiological features and blood tests were recorded when available.

### 2.3. Statistical Analysis

Statistical analysis of associations between demographic, clinical, familial, or radiological characteristics and each specific mutation was performed using IBM SPSS (version 20). Continuous data were compared using two-tailed t-tests. Comparison of categorical data was performed by means of Pearson’s Χ2 test (or Fisher’s exact test, if appropriate). The threshold for statistical significance was *p* < 0.05.

## 3. Results

We identified 40 publications fulfilling the inclusion criteria and collected information on 58 cases. The demographic data are summarized in Table 1. In order to explore possible genetic risk factors linked to CBS, we also reviewed eight publications that mainly dealt with the *MAPT* H1c haplotype and *MOBP* (myelin-associated oligodendrocyte basic protein).

*GRN* was the most common gene involved, with 28 cases (48%) from 23 families, followed by *MAPT* (16%), *C9ORF72* (10%), and *PRNP* (7%) (Table 1).

As many as 55 patients had a clearly recorded age at onset, and for 16 of them, the age at death was also available; however, gender was only indicated for 47 patients.

Considering the study patient population as a whole, the most frequently reported symptoms/signs were asymmetric akinetic–rigid motor syndrome (97%), limb or orobuccal apraxia (71%), cognitive impairment (71%), language difficulties (52%), frontal lobe syndrome/executive impairment (52%), behavioral changes (45%), and dystonia (41%).

In 24 cases (41.4%), the left side was the most affected, whereas 13 patients (22.4%) reported symptoms mainly on the right side; for the remaining 21 cases (36.2%), it was either not possible to define a predominantly affected side or the data were unavailable.

In 70% of the cases, a positive family history was reported for one of the following neurological diseases: CBS, Parkinson’s disease (PD), frontotemporal dementia (FTD), or non-specific dementia.

### 3.1. CBS Caused by a Single-Gene Mutation

#### 3.1.1. GRN Mutations in CBS

The *GRN* gene (chr17q21.32) consists of 12 coding exons and one non-coding exon. It encodes progranulin, a secreted glycoprotein that is proteolytically cleaved to form granulin (*GRN*) peptides [13].

Granulins are involved in various cellular processes, such as inflammation, lysosomal function, cell cycle, and tissue repair [14], acting as tumor progression factors [15]. They also modulate the turnover of several proteins, including TAR DNA-binding protein 43 (TDP-43) [16].

In the central nervous system, *GRN* carries out a neurotrophic activity and is highly expressed in the granule cells of the hippocampus and in the Purkinje cells of the cerebellum [17].

Most pathogenic alterations in *GRN* gene are either frameshift or nonsense mutations causing premature termination of the coding sequence and degradation of the mutant RNA by nonsense-mediated decay [18]. However, point mutations disrupting the structure of the protein are also strongly suspected to be pathogenic [19]. Loss-of-function mutations in the *GRN* gene cause haploid insufficiency of progranulin, whose plasma levels decrease as a consequence [20,21].

Autosomal dominant *GRN* mutations are a major cause of FTD [22] with ubiquitin and TDP-43-immunoreactive and tau-negative neuronal inclusions [23], but are also associated with other clinical phenotypes [24,25], such PSP and CBS [26,27]. Consequently, the clinical and pathological overlap between FTD and other parkinsonian disorders has led to the concept of a tauopathies spectrum. Moreover, homozygous *GRN* mutations have also been reported in cases of neuronal ceroid lipofuscinoses type 11 [28,29].

In the present review of published CBS cases, *GRN* turned out to be the most common causative gene, occurring in 48% of CBS cases. All *GRN* mutations were either insertions or deletions, causing frameshift, except for one missense mutation (*GRN* A199V) that was described as possibly pathogenetic [30].

Positive family history for neurologic disorders was present in 61% of cases, of which 50% showed a similar CBS phenotype [31]; in 32% of familial cases, a diagnosis of FTD in at least one other member of the family was reported.

The mean age at onset in *GRN* mutation carriers was 58.0 years (range: 43–70 years), which is similar to that for sporadic CBS.

A significant association was found between the presence of *GRN* mutations and specific clinical manifestations shared with FTD, including visuospatial impairment, behavioral changes, aphasia, and language alterations (Table 2). Indeed, several articles reported language dysfunction as a presenting symptom in *GRN* carriers [32,33]. Therefore, *GRN* mutations seem to predispose to a neurocognitive profile that later evolves into two possible phenotypes—FTD or CBS.

The frequency of core features typical of sporadic CBS, such as asymmetrical akinetic parkinsonism, apraxia, myoclonus, pyramidal signs, cognitive impairment, cortical sensory loss, frontal lobe syndrome, and alien-limb phenomenon, was not significantly different between *GRN* and non-*GRN* subgroups (Table 2).

Concerning the MRI data, 14 *GRN* carriers showed asymmetrical cortical atrophy contralateral to the most involved clinical side, mainly involving the fronto-temporo-parietal cortex [34,35,36], while only one case exhibited symmetrical diffuse cortical atrophy [37]. Despite the lack of imaging data for 13 *GRN* patients, these observations are concordant with those of previous studies, demonstrating that *GRN* mutations are associated with widespread and asymmetric atrophy that concerns the frontal, temporal, and parietal lobes [38,39].

The neuropathologic examination was available for seven subjects only [40,41]; all of them showed TDP-43 and ubiquitin-positive, tau-negative inclusions that were most prominent in the frontal and parietal cortices and, to a lesser extent, in the temporal cortex, hippocampus, and basal ganglia, which is compatible with the FTLD-U pattern [42]. In three cases, the presence of cytoplasmatic and intranuclear inclusions predominant in the second cortical layer was reported; these characteristics are typically associated with *GRN* mutation and correspond to FTLD-TDP pathology type 3 according to Sampathu et al. [43] or to type 1 in the classification proposed by Mackenzie et al. [44].

#### 3.1.2. MAPT Mutations in CBS

The *MAPT* gene (chr17q21.1) encodes microtubule-associated protein tau, whose main function is the modulation of microtubule architecture and spatial arrangement of cell structures [45].

*MAPT* mutations prevent the binding of tau protein to microtubules and lead to the synthesis of isoforms that are susceptible to hyperphosphorylation and precipitation as insoluble fibrillar aggregates, which are also known as tangles [46]. The predominant aggregation of four-repeat (4R tau) or three-repeat (3R tau) isoforms as a consequence of exon 10 splicing led to different neurodegenerative patterns.

Mutations in the *MAPT* gene inherited as an autosomal dominant trait are strongly associated with tauopathies. They are an established cause of FTD with parkinsonism-17 (FTDP-17) [47] and, less frequently, of PSP [48]. In rare cases, certain MAPT mutations can lead to a CBS presentation [49,50,51,52,53,54,55].

Nine cases of *MAPT* carriers from seven families presenting as CBS have been reported. Pathogenic *MAPT* mutations implicated in CBS are only missense variants, with the most frequent being P301S, which was identified in three patients.

The mean age at onset in carriers of *MAPT* mutations was 48.2 years (range: 27–70 years), which is slightly lower than the mean age at onset for CBS.

A positive family history of parkinsonism or dementia resulted extremely frequent in *MAPT* mutation carriers (7 out of 9 subjects, 78% of the total *MAPT* subgroup), confirming *MAPT* mutations as highly penetrant [56].

**Figure 1:**
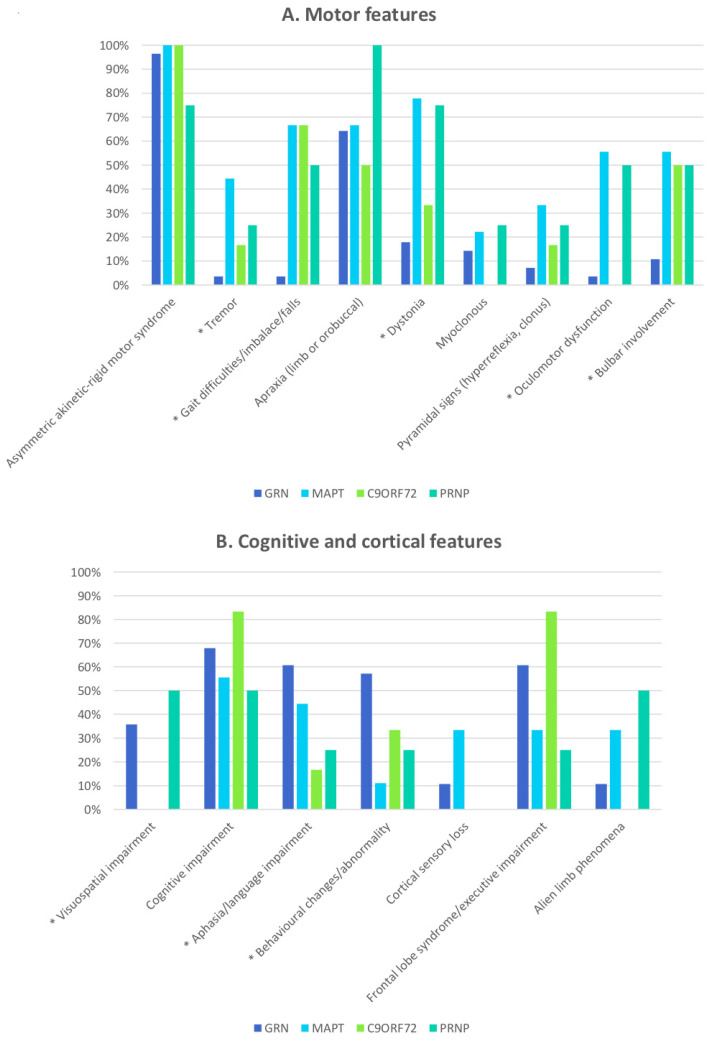
Histograms showing the frequency of motor (**A**) and cognitive (**B**) features in patients with CBS associated with mutations in *GRN*, *MAPT*, *C9ORF72*, and *PRNP*. The asterisk (*) marks clinical features that were statistically different between *GRN* patients and non-*GRN* cases, as reported in Table 2.

The clinical description of *MAPT* cases highlighted the pervasive presence of motor impairment, showing the highest prevalence of tremor (both resting and postural), dystonia, and oculomotor dysfunction (vertical upgaze limitation and slowing of saccades were the main findings) among the different subgroups of patients (Figure 1A).

Memory, language, and cortical sensory functions were also often impaired in *MAPT* carriers (Figure 1B).

The neuroimaging data that were available for seven *MAPT* mutation carriers showed bilateral frontal and/or parietal atrophy with only mild asymmetry in four patients. In contrast, three cases presented marked asymmetric atrophy of the fronto-temporo-parietal areas. In two other patients, brain imaging was not available. Due to the small sample size and the variability of the MRI findings, it was not possible to identify a common neuroradiological pattern in *MAPT* carriers, who are generally reported to show a predominant anteromedial temporal lobe atrophy, which is often bilateral [57].

Two patients underwent 123I ioflupane SPECT, revealing an asymmetrically reduced signal in the basal ganglia as in sporadic CBS.

Finally, two cases had confirmed CBD pathology by autopsy, with four-repeat-tau positive inclusions in neurons and glia as well as marked diffuse gliosis.

#### 3.1.3. C9ORF72 Expansion in CBS

The *C9ORF72* gene (chr9p21.2) encodes the *C9ORF72* protein, which is located mainly in the cytoplasm of neurons as well as in presynaptic terminals. *C9ORF72*-like proteins are thought to regulate endosomal trafficking [58], nucleocytoplasmic transport [59], and RNA processing [60].

*C9ORF72* contains an intronic hexanucleotide repeat (GGGGCC), whose expansion (>30 repeats) is the most common cause of familial autosomal dominant amyotrophic lateral sclerosis (ALS) and FTD with ALS [61]. Parkinsonian features have been reported in up to 35% of patients with *C9ORF72* mutations [62].

Six unrelated cases harboring the *C9ORF72* expansion displayed a phenotype presenting as CBS [63,64,65,66]. The mean age at onset was 50.2 years (range: 42–60 years). Positive family history for dementia was reported in all cases except one.

The clinical manifestations differentiating *C9ORF72* patients from CBS cases carrying other mutations were the frequency and predominance of cognitive impairment and frontal dysexecutive syndrome (Figure 1B), manifesting with disinhibition, apathy, attention deficit, mutism, hyperphagia, and rituals.

Of the six reported patients, two showed asymmetric frontotemporal atrophy on MRI, and two presented generalized cortical atrophy; imaging was unavailable in the other two cases.

Evidence from the literature shows that *C9ORF72* is associated with widespread brain atrophy that progresses with an antero-posterior gradient over time and involves the cerebellum [38].

None of the six cases underwent a neuropathological examination.

#### 3.1.4. PRNP Mutations in CBS

Corticobasal syndrome can occur as an early manifestation in sporadic and genetic Creutzfeldt–Jakob disease (gCJD) [67,68,69].

gCJD presenting with CBS carries an autosomal dominant mutation of the prion protein (*PRNP*) gene, with E200K being the most common [70]. *PRNP* mutations cause the conversion of the encoded protein from the normal PrPC into the protease-resistant form, called PrPRes, which aggregates in the brain and destroys neuronal tissue [71].

We found four unrelated cases with gCJD presenting as CBS. The age at onset and the age at death were, respectively, 58.8 years (range 43–73) and 60.5 years (range 45–73) on average.

The clinical manifestations were similar to the CBS phenotype at the onset, but showed a rapidly progressive course, and all patients died within 1–2 years since the onset of the disease.

Only one subject had a positive family history of CJD.

Apraxia, myoclonus, and visuospatial impairment seem more frequent in this group, but this finding did not reach statistical significance, probably because of the small sample size. Similarities were seen between CBS patients with *PRNP* and *MAPT* mutations, with high recurrence in extrapyramidal symptoms, dystonia, oculomotor and bulbar dysfunction, cognitive impairment, and alien-limb phenomena (Figure 1).

MRI findings, when available, were essential for supporting the final diagnoses; in fact, cortical and deep gray matter hyperintensity on T2 and cortical ribboning on DWI were strongly suggestive of prion disease in at least two of the reported cases.

The neuropathology was available in three cases. Two revealed a typical prion pattern with spongiosis, neuronal loss, astrogliosis, and immunohistochemically detection of pathological prion protein. On the other hand, in a subject described by Jung et al. [72], the autopsy proved a CBD, with a neuronal tau pathology both in the cortex and in the basal ganglia. The genetic analysis did not reveal any mutation in the *MAPT* or *GRN* genes; instead, a homozygous 24 bp deletion in the octapeptide repeat region in the *PRNP* gene was detected.

#### 3.1.5. Other Genes

One case of CBS characterized by asymmetric akinetic-rigid syndrome, limb dystonia, apraxia, and cognitive/behavioral changes was described in a patient affected by type 1 Gaucher’s disease [73] with compound heterozygous *GBA* mutations (N370S/L444P). Pilotto et al. [74] screened the most common *GBA* mutations in 39 patients with a clinical diagnosis of probable CBS and found two patients with a heterozygous N370S variation. These findings are interesting since they indicate that N370S *GBA* mutations may cause heterogeneous clinical phenotypes, aside from PD and dementia with Lewy bodies.

A unique family affected by cerebrotendinous xanthomatosis due to autosomal recessive mutations in CYP27A1 presented with asymmetric parkinsonism, apraxia, and dystonia, resembling a CBS phenotype [75].

In a Belgian study, mutation analysis of *CHMP2B* was performed in 134 FTLD patients, seven CBS patients, and five PSP patients. A missense mutation in exon 5 of *CHMP2B* (N143S) was found in a single patient with CBS [76].

In an extensive screening for *LRRK2* mutations [77], among eight patients found to carry the common G2019S mutation, one had previously received a diagnosis of CBS. *LRRK2* autosomal dominant mutations are associated with typical forms of PD, and only a few exceptions reporting different phenotypes exist in literature [78]. However, the G2019S mutation has been reported in some cases with tau-predominant pathology [79], suggesting a possible association with different neurodegenerative disorders as well.

Navarro et al. [80] described a family with pathologically confirmed cases of early-onset Alzheimer’s disease linked to a mutation of the presenilin-1 gene (*PSEN-1*); one member of the family developed an atypical phenotype, with left-upper-limb bradykinesia and dystonia, myoclonus, apraxia confined to the left limbs, hemispatial neglect, and frontal dementia, thus mimicking CBS. The clinical features were coherent with the MRI findings, showing severe asymmetric cortical atrophy (right more than left).

Recently, a case of probable CBS was described for the first time in a patient with an *APP* mutation and a very relevant positive family history for dementia, parkinsonism, and behavioral disorders [81]. In this patient, as in the previous one with *PSEN-1* mutation, CBS was probably underpinned by AD pathology, since mutations in APP, PSEN1, and PSEN2 are the pathogenic cause of autosomal dominant AD.

In a large study evaluating *FUS* and *TARDBP* (i.e., TDP-43) mutations in 158 FTD and 70 CBS patients, the authors identified the *TARDBP* N267S mutation in a CBS patient. Additionally, the sequencing analysis of *FUS* revealed a heterozygous insertion in exon 5 Gly175-Gly176 ins GG in a patient diagnosed with CBS, but this variant was also found among normal controls, making it thus unlikely to be pathogenetic [82].

Finally, two first cousins were reported as having asymmetric parkinsonism, apraxia, myoclonus, dystonia, cortical sensory loss, and ataxic gait, which clearly resembled CBS [83]. Post-mortem examination of both patients was consistent with CBD. Since genetic tests for *FXTAS*, *GRN*, *MAPT*, and *LRRK2* mutations were negative, both underwent exome sequencing, which revealed mutations in *MRS2* and *ZHX2* genes, and were predicted as possibly pathogenic. *MRS2* encodes for a Mg2+ channel, while the *ZHX2* gene product acts as a transcriptional regulator implicated in neuronal differentiation. The roles of *MRS2* and *ZHX2* in CBD remain to be further investigated.

### 3.2. Genetic Risk Factors Associated with CBS and CBD

So far, two major genetic risk factors have been found to be associated with CBS: homozygosity for the *MAPT* H1c haplotype and *MOBP* (myelin-associated oligodendrocyte basic protein).

The H1 *MAPT* haplotype confers a higher risk of developing both PSP (odds ratio of 5.5) and CBS/CBD (odds ratio of 3.7) [84,85,86]. Interestingly, the H1 haplotype increases the transcription of four-repeat tau isoforms [87], which are more prone to aggregation, leading to the characteristic anatomopathological stigmata of tauopathies. Litvan et al. [88] also highlighted that the H1/H1 genotype was associated with a worse motor function in terms of Unified Parkinson’s Disease Rating Scale III scores; they also described a trend toward shorter survival in the H1/H1 group, but the study was underpowered in finding a statistically significant influence on survival.

However, the role of the H1/H1 tau genotype remains unclear, since it is also common in the general population (76.6%) [89].

Considering risk factors other than *MAPT*, Kouri et al. [90] performed a genome-wide association study (GWAS) of 152 pathologically confirmed CBD cases and identified SNPs at 3p22 *MOBP*, showing another genetic risk factor shared by CBD and PSP. No clinical information was reported; therefore, it is impossible to establish whether those cases displayed a CBS phenotype.

Similarly, Yokoyama et al. [91] identified novel genetic overlap among CBD, PSP, and FTD within loci representing *CXCR4* (C-X-C chemokine receptor type 4), *EGFR* (epidermal growth factor receptor), and *GLDC* (glycine decarboxylase). The authors suggested that these genes may influence the regional pattern of volume loss and, thus, the disease phenotype; for instance, patients with *MAPT* and *CXCR4* susceptibility variants may have a higher risk of developing PSP, while patients with *MAPT*, *MOBP*, and *GLDC* may be at greater risk for CBD/CBS or FTD than PSP.

Finally, an Italian study [92] suggested that the vascular endothelial growth factor (*VEGF*) A-G-G haplotype confers an increased risk of developing tauopathies, as it is overrepresented in both PSP (OR = 6.64) and CBS (OR = 5.20), as well as FTD.

## 4. Discussion

**Figure 2:**
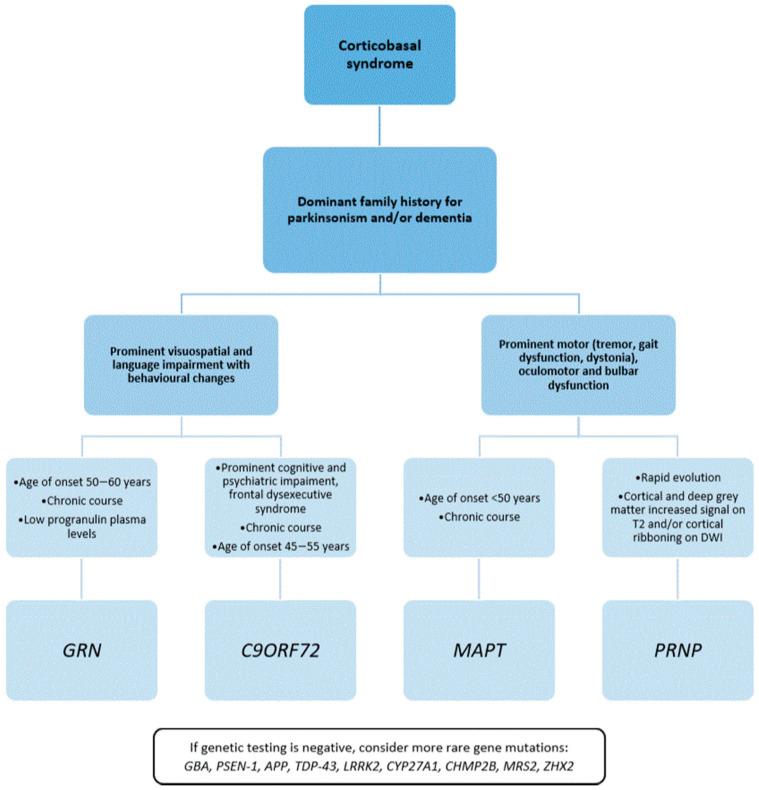
Diagnostic algorithm for screening genetic causes of CBS.

Several genetic conditions may phenotypically present as CBS, but usually show additional features that may provide important clues for the genetic diagnosis. We integrated these features into a diagnostic algorithm and proposed a three-step diagnostic approach (Figure 2) according to the findings.

First, an accurate family health history collection is of great importance. As CBS is more frequently sporadic, the presence of other affected family members represents the first hint in screening genetic forms.

A dominant family history of adult-onset parkinsonism or dementia would point mainly towards *GRN*, *MAPT,* and *C9ORF72*; a recessive pattern of inheritance or a more complex neurological phenotype may suggest other diseases less frequently associated with CBS-like phenotypes, such as cerebrotendinous xanthomatosis. A pedigree that reports PD or Gaucher disease may raise the suspicion of *GBA* mutations. Importantly, a positive family history for psychiatric disorders and motor neuron disease may lead to prioritization of *C9ORF72* mutation screening.

Notably, a negative family history does not rule out genetic disorders and may be due to reduced penetrance, variable age at onset, or de novo mutations.

Secondly, some distinct clinical features may suggest a specific mutation (Table S1).

*GRN*, *C9ORF72*, and *PSEN-1* carriers seem to have predominant cortical involvement, clinically presenting with visuospatial and language impairment and behavioral changes. Conversely, *MAPT* and *PRNP* carriers more frequently develop motor symptoms suggestive of subcortical and brainstem areas, such as dystonia, tremor, gait dysfunction, and oculomotor and bulbar dysfunction. Therefore, according to the more affected function, i.e., cognitive or motor, a different subset of mutations should be taken into consideration.

As a third point, features to be considered are the age of onset and the disease course.

The mean age at onset of sporadic CBS is from 50 to 60 years; thus, a younger age at the onset of symptoms should suggest a genetic form of CBS.

According to available evidence, there is a gradient in the symptom onset age according to the specific causative mutation: *MAPT* carriers have an earlier onset, followed by *C9ORF72* and *GRN* carriers [93]. Our review confirms these findings and suggests considering *MAPT* mutations in patients with a clinical onset before the age of 50.

The clinical course of sporadic CBS is that of a chronic neurodegenerative condition with a median survival of 6 to 7 years. Thus, a more rapid progression should be considered a red flag and may suggest a prion disease or an acquired condition, such as autoimmune or vascular disorders [94].

Lastly, biochemical and radiological data may support the diagnosis. The evaluation of plasmatic progranulin levels is useful for raising the suspicion of a *GRN* mutation [95] and should be performed even in the absence of positive family history for movement disorders.

EEG showing periodic sharp-wave complexes and MRI results displaying gray matter hyperintensity on T2 and cortical ribboning on DWI are strongly suggestive of prion disease.

A single genetic mutation may not be sufficient for determining a CBS phenotype, which likely requires additional hits. Gene expression is influenced by modulation of genetic and environmental factors, whose effect can induce, for example, the expression of a *GRN* mutation as CBS rather than FTD. Further studies exploring whole-genome variants of individuals with one of the mutations also described in CBS may shed light on the specific genetic architecture underling CBS.

Identifying genetic variants underlying CBS has a great practical relevance, since it allows appropriate family counseling and the possibility to be enrolled in clinical trials with disease-modifying treatments. This process is currently ongoing for *GRN* and *GBA* mutation carriers, but will likely be extended to other gene variants in the future.

Finally, considering the data from the literature reported here and prompted by our and others’ experience of CBS cases with GBA or AD gene mutations [74,81], we strongly encourage to screen CBS patients for genes correlated with other neurodegenerative diseases when more frequent mutations have been excluded.

## 5. Conclusions

The knowledge of the genetic background of CBS is expanding rapidly, and it helps to improve diagnostic accuracy and the understanding of the underlying molecular pathogenetic processes.

Positive family history and an earlier age at onset are the two main clues that should lead clinicians to suspect and test for genetic conditions. However, an age at onset comparable with that of sporadic cases should not rule out the possibility of genetic testing, especially if *GRN* is suspected.

Our review of genotype–phenotype correlation in corticobasal syndrome emphasizes the importance of a complete and in-depth clinical assessment. In fact, although there is a significant overlap in terms of clinical presentation, there may be some demographical, clinical, biochemical, and radiological features that are significantly evocative of mutations in specific genes.

## Figures and Tables

**Table 1:** Demographic information of genetic corticobasal syndrome (CBS) cases included in the analysis. * indicates that values were taken only where available.

	Number of Cases (% of Total Cases)	Mean Age of Onset * (Years)	Mean Age at Death * (Years)	Male *	Female *
*GRN*	28 (48%)	58.08	63.57	10	7
*MAPT*	9 (16%)	48.22	56.33	4	5
*C9ORF72*	6 (10%)	50.17	NA	2	4
*PRNP*	4 (7%)	58.75	60.5	1	3
*GBA*	3 (5%)	62.6	NA	1	2
*MRS2/ZHX2*	2 (3%)	71	74	1	1
*PSEN-1*	1 (<2%)	48	51	1	0
*APP*	1 (<2%)	49	NA	0	1
*TDP-43*	1 (<2%)	72	NA	1	0
*CHMP2B*	1 (<2%)	71	NA	0	1
*LRRK2*	1 (<2%)	52	NA	0	1
*CYP27A1*	1 (<2%)	47	NA	1	0
Total	58	56.2 (SD 10.45)	61.31 (SD 9.85)	22	25

**Table 2:** Comparison between *GRN* and *MAPT/C9ORF72* carriers. Statistically significant results are in bold; the threshold for statistical significance was *p* < 0.05.

	*GRN*	*MAPT/C9ORF72*	*p* Value
Number of patients	28	15	−
Gender, male *(GRN 17; MAPT 9; C9ORF72 6)*	58.8% (10)	40.0% (6)	0.288
Age at disease onset, years *(GRN 25; MAPT 9; C9ORF72 6)*	58.1 ± 8.1	49.0 ± 11.2	**0.005**
Asymmetric akinetic–rigid syndrome *(GRN 28; MAPT 9; C9ORF72 6)*	96.4% (27)	100.0% (15)	0.999
Apraxia *(GRN 24; MAPT 9; C9ORF72 6)*	75.0% (18)	60.0% (9)	0.323
Gait dysfunction *(GRN 28; MAPT 9; C9ORF72 6)*	3.6% (1)	66.7 % (10)	**<0.001**
Tremor *(GRN 24; MAPT 9; C9ORF72 6)*	4.2% (1)	33.3% (5)	**0.024**
Dystonia *(GRN 24; MAPT 9; C9ORF72 6)*	20.8% (5)	60.0% (9)	**0.013**
Myoclonus *(GRN 24; MAPT 9; C9ORF72 6)*	16.7% (4)	13.3% (2)	0.999
Pyramidal signs *(GRN 24; MAPT 9; C9ORF72 6)*	8.3% (2)	26.7% (4)	0.180
Oculomotor dysfunction *(GRN 23; MAPT 9; C9ORF72 6)*	4.3% (1)	33.3% (5)	**0.027**
Bulbar involvement *(GRN 28; MAPT 9; C9ORF72 6)*	10.7% (3)	53.3% (8)	**0.004**
Aphasia/language impairment *(GRN 24; MAPT 9; C9ORF72 6)*	70.8% (17)	33.3% (5)	**0.022**
Visuospatial impairment *(GRN 20; MAPT 9; C9ORF72 6)*	50.0% (10)	0% (0)	**0.002**
Cognitive impairment *(GRN 23; MAPT 9; C9ORF72 6)*	82.6 % (19)	66.7% (10)	0.436
Behavioural changes *(GRN 24; MAPT 9; C9ORF72 6)*	66.7% (16)	20.0% (3)	**0.008**
Frontal lobe syndrome *(GRN 23; MAPT 9; C9ORF72 6)*	73.9% (17)	53.3% (8)	0.191
Cortical sensory loss *(GRN 24; MAPT 9; C9ORF72 6)*	12.5% (3)	20.0% (3)	0.658
Alien limb *(GRN 24; MAPT 9; C9ORF72 6)*	12.3% (3)	20.0% (3)	0.658

## Supplementary Materials

The following are available online at https://www.mdpi.com/2073-4409/10/1/171/s1, Table S1: Analysis of clinical features of *GRN*, *MAPT* and *C9ORF72* carriers.
