# Supplementary material for: Unravelling Genetic Factors Underlying Corticobasal Syndrome: A Systematic Review
Source: Cells. 2021 Jan 15;10(1):171. doi: 10.3390/cells10010171 (PMC7830591; doi:10.3390/cells10010171)
Supplement: Supplementary file 1 [file cells-10-00171-s001.pdf]

	PRGN	MAPT	C9ORF72	*p* value *GRN* vs *MAPT*	*p* value *GRN* vs *C9ORF72*
Number of patients	28	9	6	–	–
Gender, male *(GRN 17; MAPT 9; C9ORF72 6)*	58.8% (10)	44.4% (4)	33.3% (2)	0.683	0.371
Age at disease onset, years *(GRN 25; MAPT 9; C9ORF72 6)*	58.1 ± 8.1	48.2 ± 13.8	50.2 ± 6.6	**0.015**	**0.034**
Asymmetric akinetic-rigid syndrome *(GRN 28; MAPT 9; C9ORF72 6)*	96.4% (27)	100.0% (9)	100% (6)	0.999	0.999
Apraxia *(GRN 24; MAPT 9; C9ORF72 6)*	75.0% (18)	66.7 % (6)	50.0% (3)	0.677	0.329
Gait dysfunction *(GRN 28; MAPT 9; C9ORF72 6)*	3.6% (1)	66.7 % (6)	66.7% (4)	**< 0.001**	**0.002**
Tremor *(GRN 24; MAPT 9; C9ORF72 6)*	4.2% (1)	44.4% (4)	16.7% (1)	**0.013**	0.366
Dystonia *(GRN 24; MAPT 9; C9ORF72 6)*	20.8% (5)	77.8% (7)	33.3% (2)	**0.005**	0.603
Myoclonus *(GRN 24; MAPT 9; C9ORF72 6)*	16.7% (4)	22.2% (2)	0% (0)	0.999	0.557
Pyramidal signs *(GRN 24; MAPT 9; C9ORF72 6)*	8.3% (2)	33.3% (3)	16.7% (1)	0.111	0.501
Oculomotor dysfunction *(GRN 23; MAPT 9; C9ORF72 6)*	4.3% (1)	55.6% (5)	0% (0)	**0.003**	0.999
Bulbar involvement *(GRN 28; MAPT 9; C9ORF72 6)*	10.7% (3)	55.6% (5)	50.0% (3)	**0.012**	0.053
Aphasia/language impairment *(GRN 24; MAPT 9; C9ORF72 6)*	70.8% (17)	44.4% (4)	16.7% (1)	0.230	**0.026**
Visuospatial impairment *(GRN 20; MAPT 9; C9ORF72 6)*	50.0% (10)	0% (0)	0% (0)	**0.011**	0.053
Cognitive impairment *(GRN 23; MAPT 9; C9ORF72 6)*	82.6 % (19)	55.6% (5)	83.3% (5)	0.176	0.999
Behavioural changes *(GRN 24; MAPT 9; C9ORF72 6)*	66.7% (16)	11.1% (1)	33.3% (2)	**0.007**	0.184
Frontal lobe syndrome *(GRN 23; MAPT 9; C9ORF72 6)*	73.9% (17)	33.3% (3)	83.3% (5)	**0.049**	0.999
Cortical sensory loss *(GRN 24; MAPT 9; C9ORF72 6)*	12.5% (3)	33.3% (3)	0% (0)	0.309	0.999
Alien Limb *(GRN 24; MAPT 9; C9ORF72 6)*	12.3% (3)	33.3% (3)	0% (0)	0.309	0.999

Supplementary Table 1. Analysis of clinical features of *GRN*, *MAPT* and *C9ORF72* carriers. Comparisons have been made between *GRN* vs *MAPT* and *GRN* vs *C9ORF72* cases. Statistically significant results are highlighted; threshold for statistical significance was $p < 0.05$.
